# The Role of Protein Post-Translational Modifications in the Pathogenesis of Nephrolithiasis: Mechanistic Insights and Translational Potential

**DOI:** 10.3390/cells15060554

**Published:** 2026-03-19

**Authors:** Wenlong Wan, Baokang Wang, Junyi Yang, Yang Xun, Xiao Yu

**Affiliations:** Tongji Hospital, Tongji Medical College, Huazhong University of Science and Technology, Wuhan 430074, China; d202482550@hust.edu.cn (W.W.);

**Keywords:** protein post-translational modifications, nephrolithiasis, phosphorylation, acetylation, crystal-cell interactions

## Abstract

**Highlights:**

**What are the main findings?**

- Protein post-translational modifications (PTMs) such as phosphorylation, acetylation, and ubiquitination act as central “molecular switches” that orchestrate crystal-cell adhesion, oxidative stress, inflammatory signaling, and diverse programmed cell death pathways (ferroptosis, pyroptosis, necroptosis) in nephrolithiasis.
- The kidney stone microenvironment (hyperoxaluria, oxidative stress, metabolic reprogramming) actively shapes the PTM landscape of key proteins, creating a complex regulatory network that determines the balance between renal injury and repair.

**What is the implication of the main finding?**

- Targeting specific PTM-regulating enzymes (e.g., Sirt1 activators, HDAC2 inhibitors, AMPK activators) represents a promising novel therapeutic strategy to interrupt stone formation and halt disease progression.
- Deciphering the “PTM code” that integrates microenvironmental signals to dictate cell fate decisions provides a new framework for understanding stone pathogenesis and developing precision intervention strategies.

**Abstract:**

Nephrolithiasis is a prevalent urological disorder worldwide, whose pathogenesis involves a complex network of crystal formation, cellular injury, and microenvironmental dysregulation. As a critical mechanism for regulating cellular functions, protein post-translational modifications (PTMs) have been increasingly implicated in multiple facets of kidney stone formation, including crystal–cell interactions, oxidative stress responses, and inflammatory signaling pathways. This review systematically synthesizes the biochemical foundations of PTMs, the molecular microenvironment of nephrolithiasis, and the roles of key modifications such as phosphorylation and acetylation in the pathogenesis of calculi. It further explores the translational potential of PTM detection technologies in clinical practice. Current evidence indicates that PTMs influence the nucleation, growth, and aggregation of crystals by modulating the activity of pro-/anti-lithogenic proteins, the expression of cell adhesion molecules, and inflammatory pathways. Consequently, therapeutic strategies targeting PTMs may offer novel avenues for the prevention and management of kidney stones. Future research should focus on integrating multi-omics approaches with functional validation to elucidate the dynamic regulatory networks of PTMs within the stone microenvironment, thereby advancing the development of precision medicine.

## 1. Introduction

Nephrolithiasis stands as one of the most prevalent urological disorders globally, with its prevalence and incidence demonstrating a significant upward trend [1,2]. This condition is characterized by a high recurrence rate, imposing repeated episodes of pain and a diminished quality of life on patients, alongside placing a substantial economic burden on healthcare systems worldwide [3,4]. Beyond being an isolated urological ailment, nephrolithiasis is increasingly recognized as a systemic condition associated with an elevated risk of various comorbidities, including chronic kidney disease (CKD), cardiovascular diseases, and metabolic syndrome.

The traditional view of nephrolithiasis pathogenesis centers on a physicochemical “supersaturation-crystal formation-retention” process. This paradigm posits that stone formation initiates when urinary concentrations of lithogenic substances exceed their solubility, leading to crystal formation. These crystals subsequently become retained within the kidney, where they grow, aggregate, and ultimately evolve into clinically detectable stones [5,6,7]. However, this classic theory fails to fully explain all clinical observations, such as why not all individuals with supersaturated urine develop stones, and the precise underlying mechanisms [8,9]. With advances in imaging, molecular biology, and multi-omics technologies, the academic understanding of stone formation has evolved. The paradigm has shifted from a purely physicochemical model to a recognition of nephrolithiasis as a complex biological event. This new perspective encompasses multiple dimensions and pathways involving cell biology, immune inflammation, the microbiome, and genetic susceptibility, which is crucial for developing novel prevention and treatment strategies [10,11].

**Figure 1:**
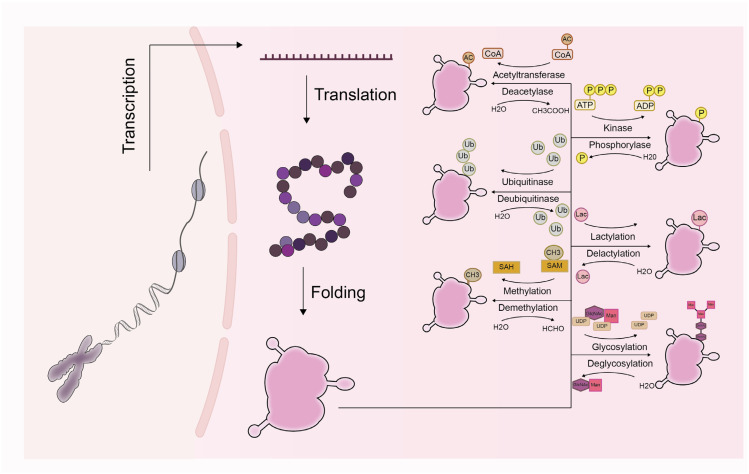
Common Protein Post-translational Modifications and Their Reverse Processes. Colored shapes represent modification groups or cofactors, and arrows indicate the direction of reactions.

Protein post-translational modifications (PTMs) refer to key regulatory mechanisms wherein chemical groups are covalently attached to amino acid residues after protein biosynthesis, thereby altering protein properties [12,13]. This process vastly expands the functional diversity of the proteome [14]. More than 650 types of PTMs have been identified, with major examples including phosphorylation, ubiquitination, glycosylation, methylation, acetylation, SUMOylation, and lactylation (Figure 1). These modifications dynamically regulate nearly all core physiological processes—such as intracellular signal transduction, metabolism, gene expression, and protein trafficking—by altering protein conformation, subcellular localization, activity, stability, and interactions with other biomolecules [15,16]. The homeostasis of PTMs is vital for maintaining cellular and organismal health, whereas aberrant PTMs are closely implicated in the pathogenesis of numerous diseases, including cancers, neurodegenerative disorders, cardiovascular diseases, metabolic disorders, and kidney diseases [17,18]. For instance, in oncology, dysregulated PTMs contribute to the control of cell proliferation, immune evasion, and drug resistance [19]; in CKD, alterations in PTMs are closely linked to processes such as renal fibrosis, inflammation, and cellular senescence [20,21,22].

While the physicochemical principles of stone formation are reasonably well-characterized, the underlying molecular regulatory networks, particularly the cellular response mechanisms, remain to be fully elucidated [23]. PTMs, representing the most direct and precise mode of cellular functional regulation, may act as pivotal “molecular switches” in the “crystal–cell–matrix” interactions central to stone pathogenesis. However, research on how specific PTMs regulate the initiation and progression of nephrolithiasis remains relatively limited. Therefore, a systematic exploration of the roles PTMs play in the pathological microenvironment of kidney stones holds significant promise. It not only promises to deepen our mechanistic understanding of stone formation but may also offer novel perspectives for developing new diagnostic biomarkers and therapeutic targets.

To systematically elucidate the role of PTMs in the pathogenesis of nephrolithiasis, we have constructed a conceptual framework that integrates physicochemical processes with PTM regulation. In this framework, physicochemical processes—crystal supersaturation, nucleation, and growth—serve as the initial driving force for stone formation, while PTMs function as a regulatory hub that determines how this force is perceived, amplified, or buffered by the cellular microenvironment. Specifically, upstream stimuli (e.g., hyperoxaluria/hypercalciuria, oxidative stress, metabolic disturbances) alter the modification status of key proteins by activating or inhibiting specific PTM enzymes. These modification changes, in turn, regulate downstream effects, including crystal–cell adhesion, inflammatory responses, modes of cell death, and metabolic reprogramming, ultimately determining whether stones will form. In this process, PTMs act as key modulators rather than initial drivers—they translate physicochemical signals into a “molecular language” interpretable by cells, thereby precisely directing the cellular response to crystal stimuli.

Based on this integrated perspective, we systematically reviewed and evaluated the existing literature. A search of the PubMed and Web of Science databases was conducted for relevant studies published since 2000, focusing on the central associations between PTMs and the pathogenesis of nephrolithiasis. The inclusion criteria were (1) original research or review articles; (2) studies addressing the association between PTMs and nephrolithiasis pathogenesis; and (3) articles published in English. The exclusion criteria were (1) studies published only in abstract form; and (2) studies with low relevance to the topic. Based on the literature selected using this strategy, different sources of evidence—including in vitro cellular experiments and animal model studies—are clearly distinguished in Table 1.

## 2. Overview of the Nephrolithiasis-Associated Microenvironment and Key Proteins

The formation of kidney stones is a dynamic process occurring within a complex microenvironment shaped by the interplay of multiple pathological factors. This microenvironment extends beyond the physicochemical conditions of hyperoxaluria/hypercalciuria to encompass interactions across various levels, including oxidative stress, inflammatory responses, programmed cell death, and metabolic reprogramming. These microenvironmental factors not only directly drive crystal nucleation, growth, and adhesion but, more importantly, can profoundly influence the function and stability of relevant proteins by modulating their PTM status, thereby accelerating or inhibiting the lithogenic process (Table 1).

### 2.1. The Basic Pathophysiological Process of Kidney Stone Formation

Kidney stone formation is a complex, multi-step process that begins with a state of supersaturation of lithogenic substances in the urine [47]. When the concentration of these substances exceeds their solubility, tiny crystal cores can form and subsequently enlarge. However, crystal formation alone is insufficient to produce clinical stones. Under normal physiological conditions, microcrystals are effectively cleared by the flushing effect of urine flow and the presence of crystallization inhibitors in the tubular fluid [48]. When renal tubular epithelial cells are damaged, adhesion molecules exposed on the cell surface can capture these crystals, allowing them to retain, grow, and ultimately form clinically detectable stones [49]. Therefore, kidney stone formation results from the interplay between physicochemical processes (crystal supersaturation, nucleation, and growth) and biological processes (cellular injury, adhesion, and inflammation) [50].

### 2.2. Influence of the Stone Microenvironment on Protein Modification Status

(1) A hyperoxaluric/hypercalciuric environment is a hallmark feature of the nephrolithiasis microenvironment.

Calcium oxalate (CaOx) crystals can directly injure renal tubular epithelial cells, inducing endoplasmic reticulum (ER) stress [51]. Proteomic network analyses indicate that stone-associated proteins are significantly enriched in ER-related processes involving PTMs [52]. The unfolded protein response triggered by crystal stimulation relies on proper PTMs to restore ER homeostasis. Dysregulation or failure of these PTMs can lead to apoptosis, thereby providing sites for crystal adhesion [53,54]. High-oxalate stimulation also induces significant changes in the expression of numerous proteins involved in pathways such as oxidative stress, apoptosis, and biomineralization, whose functions are likely regulated by corresponding PTMs [55]. Furthermore, hyperoxaluria may reduce the generation of endogenous hydrogen sulfide (H_2_S). H_2_S acts as a gaseous signaling molecule mediating protein persulfidation, and its reduction could indirectly impair the injury-protective and anti-inflammatory functions of relevant proteins [56].

(2) Oxidative Stress and Protein Oxidative Modifications

Oxidative stress is a central, persistent component of stone formation [57]. Excessive reactive oxygen species (ROS) generated can directly attack proteins, leading to oxidative modifications that alter protein structure, activity, and function [58]. For instance, cadmium exposure accelerates CaOx stone formation and renal injury by upregulating thioredoxin-interacting protein, driving the “oxidative stress–inflammation–apoptosis” axis [59]. A recent study further confirmed that the proportion of oxidatively modified proteins is significantly increased in the urine of stone formers, and these proteins are closely associated with biological processes of oxidative stress, suggesting that protein oxidative modification is a key pathogenic factor or risk marker in CaOx stone formation [60]. Notably, ROS also function as signaling molecules that can activate or inhibit various kinases and phosphatases, thereby extensively participating in the regulation of protein phosphorylation networks [61,62].

(3) Metabolic Reprogramming and Metabolite-Mediated Modifications

Metabolic dysregulation is a significant risk factor for nephrolithiasis [63]. Alterations in the levels of specific metabolites (e.g., lactate, succinyl-CoA, short-chain fatty acids) in patients may directly influence the progression of certain PTMs [64,65]. For example, succinyl-CoA is the substrate for protein succinylation; lactate levels may affect intracellular pH and the activity of deacetylases; and short-chain fatty acids can act as inhibitors or substrates for histone deacetylases [66,67]. Consequently, metabolic abnormalities within the microenvironment may drive aberrant protein modifications by altering substrate availability for PTM enzymes or the local microenvironment. These modifications can subsequently impact pathways related to inflammation, apoptosis, and fibrosis, indirectly promoting stone formation.

### 2.3. Classification of Key Proteins Involved in Stone Formation

(1) Pro-Lithogenic Proteins: These typically promote stones by binding to crystals or mediating crystal–cell adhesion.

Osteopontin (OPN) is a classical example, with its phosphorylated form exhibiting particularly potent pro-lithogenic effects [68]. OPN promotes crystal aggregation and nucleation and acts as a pro-inflammatory factor activating immune cells [69]. Studies suggest OPN levels are abnormally elevated in hyperoxaluric rat models, and treatment with 4-PBA can reverse this phenomenon [70]. Another 2025 study similarly observed that renal tubular injury and CaOx deposition in a hyperoxaluric model were associated with increased OPN secretion in urine [56]. Oncostatin M (OSM) can drive the retention of crystals on renal tubular epithelium via its receptor, promoting the expression of OPN and ANXA1/2 [71]. Similarly, the Insulin-like Growth Factor 1 Receptor (IGF1R) is involved in stone-induced renal injury processes [72]. Furthermore, Heat Shock Protein 90 (HSP90) is another critical pro-adhesive protein; it can act as a cell surface receptor for CaOx crystals, significantly enhancing cell–crystal binding [73]. Other proteins such as alpha-1-acid glycoprotein 2 and fibrinogen alpha chain have also been identified as having pro-lithogenic roles [74], and their activity may be related to specific modification states [75].

(2) Anti-Lithogenic Proteins: These inhibit crystal nucleation, growth, and aggregation.

Tamm–Horsfall Protein (THP) is a major physiological inhibitor [76]. As one of the most abundant proteins in urine, it effectively inhibits crystal aggregation. Similarly, prothrombin fragment 1 (PTF1) significantly inhibits the nucleation, growth, and aggregation of CaOx crystals, and its levels are often lower in stone formers than in healthy individuals [77]. The Sirtuin family proteins are key components of the PTM network. Notably, Sirtuin 1 (Sirt1) plays a central role in antioxidative stress, anti-inflammation, and regulating metabolic reprogramming. Its decreased expression is associated with exacerbated renal injury in stone disease [78]. Likewise, Sirtuin 6 (Sirt6) is involved in processes such as DNA damage repair, and its stability is regulated by ubiquitination [79]. Calcineurin B can directly inhibit the crystallization process of CaOx [80]. C4b-Binding Protein acts as an endogenous inhibitor of the NLRP3 inflammasome, exerting a protective role in crystal-induced inflammation [81]. The activity or stability of these proteins is also likely influenced by microenvironment-mediated PTMs.

(3) Cell Adhesion/Damage-Related Proteins: These directly mediate crystal–cell interactions.

The adhesion of crystals to renal tubular epithelial cells is a critical initiating step in stone formation, with multiple proteins participating in this process [82]. Beyond adhesion molecules like OPN and ANXA2, the cellular stress and injury responses themselves are tightly regulated by the PTM network [83]. For example, STAT3 phosphorylation not only promotes the expression of adhesion molecules but also drives the production of various pro-inflammatory and pro-fibrotic factors, further promoting stone progression [84]. Metabolically, IGF1R drives metabolic reprogramming that promotes stone-related epithelial–mesenchymal transition, exacerbating renal injury [72]. Concurrently, ANXA2 upregulation can activate the ERK1/2 and JNK signaling pathways, mediating CaOx crystal adhesion, a function potentially regulated by calcium ions and phosphorylation [85]. Additionally, certain circulating proteins have been found to be associated with stone risk [86]; they may participate in the pathological process as effectors or regulators of microenvironmental changes.

The pathological microenvironment of nephrolithiasis constitutes a complex regulatory network by influencing the PTM status of key proteins. A deeper understanding of how factors such as high oxalate, ROS, and specific metabolites regulate PTM enzyme activity and the protein modification profile will provide crucial directions for unraveling the molecular mechanisms of nephrolithiasis and identifying novel therapeutic targets.

## 3. Mechanisms of Post-Translational Modifications in Stone-Associated Renal Injury

### 3.1. PTMs in Crystal–Cell Adhesion and Initial Injury

The aberrant adhesion of crystals to renal tubular epithelial cells is the initiating step in stone formation. Various PTMs participate in this process by regulating the function of adhesion molecules and receptors [85]. A study on OPN indicated that its glycosylation status may be a key factor regulating its activity in CaOx crystal growth, aggregation, and cell adhesion [87]. Pathological signals within the microenvironment, such as elevated palmitic acid in the urine of stone formers, can promote the phosphorylation of Phosphatidylethanolamine-Binding Protein 1 via its derivative Protein Kinase C ζ. This exacerbates cell membrane lipid peroxidation, compromises membrane integrity, and thereby exposes more adhesion sites [24]. Conversely, oxalate exposure upregulates JPT2 protein, which activates the PI3K/AKT signaling pathway. This pathway has been confirmed to regulate the expression of cell adhesion-related molecules, promoting crystal–cell adhesion [25]. Furthermore, increased levels of Deoxycholic Acid (DCA) resulting from gut microbiota dysbiosis can upregulate the expression of Hsp90α on renal tubular epithelial cell membranes, directly enhancing crystal–cell adhesion capacity [40]. Beyond phosphorylation, the glycosylation state of proteins is also critical. For instance, the sialylation modification of Prothrombin Fragment 1 (PTF1) effectively promotes CaOx crystal nucleation while inhibiting its aggregation. Alterations in this protective glycosylation pattern may be associated with stone formation [45]. In metabolically abnormal environments such as hyperglycemia, cells may undergo non-enzymatic glycation of HSP90, altering its function, which could be one mechanism linking lifestyle-related diseases to increased stone risk [88]. Regarding calcium ion homeostasis, CaOx crystals can activate the Calcium-Sensing Receptor, leading to the phosphorylation of the transcription factor STAT3 by Protein Kinase A. Phosphorylated and activated STAT3 drives the expression of the tight junction protein Claudin-14, which may promote the supersaturation and deposition of stone salts by influencing the calcium concentration in the tubular lumen [26]. These diverse PTMs initiate the initial cellular injury and crystal retention by altering cell surface properties, signal transduction, and the local microenvironment, thereby providing anchor points for crystals and modifying urine chemistry.

### 3.2. PTMs in Regulating Inflammation and Oxidative Stress

Cellular inflammation and oxidative stress responses induced by CaOx crystal adhesion and injury are considered pivotal in the pathogenesis of nephrolithiasis [89], with PTMs acting as key switches in regulating these signaling pathways [90,91]. Studies suggest that the inflammatory factor Oncostatin M (OSM), induced by CaOx crystals, activates its receptor OSMRβ, leading to the phosphorylation of the transcription factor STAT3. This drives the expression of various crystal-binding molecules (e.g., OPN, ANXA1/2) and pro-inflammatory/pro-fibrotic factors, amplifying the inflammatory response and promoting fibrosis [32]. Nephrotoxic substances like oxalate or CaOx crystals can directly induce injury in renal tubular epithelial cells [92,93,94], a process closely associated with abnormally increased intracellular histone deacetylase activity. Research has shown that Histone Deacetylase 3 (HDAC3) is upregulated in stone-associated renal interstitial fibrosis. By deacetylating histones, HDAC3 suppresses the expression of miR-19b-3p, thereby lifting the inhibition on pro-fibrotic factors and promoting fibrosis progression [36]. In the protective mechanisms against oxidative stress, the deacetylase Sirtuin 1 (Sirt1) plays a crucial role [95,96]. In CaOx nephropathy, Sirt1 expression is decreased [97,98]. Activating Sirt1 can regulate metabolic enzymes via deacetylation and modulate the expression of immune-responsive gene 1 and succinate dehydrogenase through histone methylation modifications. This increases the level of itaconate, which possesses anti-inflammatory and antioxidant properties, thereby alleviating crystal deposition and renal injury [35]. Similarly, the Vitamin D Receptor (VDR) is upregulated in a genetic hypercalciuric stone-forming rat model. Upon binding to target gene promoters, VDR induces hyperacetylation or hypermethylation of histone H3 and participates in the pathogenesis of hypercalciuria [33]. Additionally, protective agents such as hyperoside and Lysimachia christinae extract can inhibit oxalate-induced oxidative stress and inflammation by promoting AMPK phosphorylation, activating the Nrf2/HO-1 antioxidant pathway, or suppressing the PI3K/Akt/mTOR pathway [27,31]. Interestingly, lactylation modifications exhibit a dual role. On one hand, lactate-mediated lactylation of the mitochondrial fission protein Fis1 can lead to excessive mitochondrial fission, ROS production, and apoptosis [99,100]. Since CaOx crystals also induce oxidative stress and mitochondrial dysfunction, lactylation likely participates in crystal-induced renal tubular epithelial cell injury [101,102]. On the other hand, appropriate lactylation might promote the expression of protective genes during the post-injury repair phase [103,104]. Therefore, dynamically monitoring and precisely intervening in lactylation balance could be a novel approach for stone prevention and treatment. Autophagy, another important protective pathway, is also regulated by PTMs. Sodium-glucose cotransporter 2 inhibitors can restore impaired autophagic flux by downregulating the activity of mammalian target of rapamycin and activating AMPK, thereby inhibiting stone formation [28]. These intricate PTM networks collectively determine the balance between inflammation and antioxidant defense, influencing the initiation and progression of nephrolithiasis.

### 3.3. PTMs Determine Cell Fate: Apoptosis, Pyroptosis, and Ferroptosis

The mode of renal tubular epithelial cell death is central to determining the extent of injury and the direction of repair, with PTMs extensively involved across different death types [105,106]. PTMs determine cell fate by differentially regulating various modes of cell death: At different stages of stone formation, the dominant mode of cell death may vary. In the acute phase, necroptosis and pyroptosis are likely predominant, mediating intense inflammatory responses. In the chronic phase, ferroptosis and apoptosis may become more prominent, contributing to tissue damage and fibrotic processes [107,108].

Ferroptosis is a regulated cell death characterized primarily by iron-dependent accumulation of lipid peroxides [109]. Studies confirm that in the stone environment, both the expression and acetylation level of p53 protein are increased. Acetylated p53 promotes ferroptosis, exacerbating CaOx crystal-induced renal fibrosis [34]. In contrast, Sirt1-mediated deacetylation of p53 can inhibit ferroptosis, exerting a protective effect [110,111]. Furthermore, DCA-upregulated Hsp90α can interact with the key antioxidant enzyme GPX4 and promote its ubiquitination and degradation, mediating lipid peroxidation accumulation and ferroptosis [40]. Conversely, von Hippel-Lindau (VHL) protein exerts a protective role by promoting K48-linked polyubiquitination and proteasomal degradation of the adaptor protein BICD2, thereby inhibiting STAT1 nuclear translocation and downstream pro-ferroptotic signaling. BRAF inhibitors can induce BICD2 phosphorylation, disrupting its binding with VHL and leading to severe ferroptosis and renal injury [41]. In pyroptosis regulation, the transcription factor KLF4 is regulated upstream by the deubiquitinase USP11 [112]. USP11 stabilizes KLF4 protein via deubiquitination, leading to its upregulation. Elevated KLF4 then directly transcriptionally activates Caspase-1 and Caspase-3, mediating cell pyroptosis via the GSDMD and GSDME pathways, respectively, thereby driving inflammation and fibrosis [43]. Regarding necroptosis, CaOx crystals can activate Receptor-Interacting Protein Kinase 3. A specific RIPK3 inhibitor effectively blocks the formation of the RIPK1-RIPK3 necrosome, suppressing necroptosis and inflammation [30]. Mitochondrial quality control is also tightly linked to cell fate [113]. Melatonin activates AMPK phosphorylation, enhancing PINK1-Parkin-mediated mitophagy, reducing ROS release, and thereby inhibiting oxalate-induced oxidative stress, inflammation, and ferroptosis, ultimately suppressing stone formation [29]. Another study on pediatric nephrolithiasis found that upregulation of PINK1 kinase expression and its mediated excessive mitophagy were highly correlated with renal tubular epithelial cell apoptosis and stone formation [114].

The regulatory mechanisms described above do not function in isolation. Instead, through a “modification code” established by PTMs, the switch and balance between different cell death pathways are precisely regulated. For instance, the acetylation of p53 promotes ferroptosis, while its phosphorylation may influence apoptosis [115]; the phosphorylation status of RIPK3 determines the activation threshold for necroptosis. This modification code integrates multiple environmental signals, including crystal stimulation, metabolic changes, and oxidative stress, ultimately dictating which death pathway a cell will undergo.

Based on this precise regulation of cell death pathways by PTMs, intervening in the modifications of key proteins—such as acetylation, ubiquitination, and phosphorylation—is emerging as a highly promising therapeutic strategy. Notably, research indicates that the aberrant activation of HDAC2 plays a critical role in renal tubular injury. Specifically inhibiting HDAC2 can effectively reduce renal tubular epithelial cell apoptosis by upregulating the expression of the protective factor BMP-7 and promoting the polarization of M1 macrophages toward the anti-inflammatory M2 phenotype [116,117,118]. This provides a compelling example of how targeting PTMs to regulate cell death modes can intervene in kidney stone-related renal injury. Future research should further elucidate how different modification codes integrate environmental signals and how the switches between various death modes are precisely regulated in a spatiotemporal manner. Such investigations will lay the theoretical foundation for developing precise intervention strategies for kidney stones and associated renal injury.

### 3.4. PTMs Mediate Metabolic Reprogramming and Adaptation

Kidney stone formation is often accompanied by significant cellular metabolic reprogramming. PTMs play important roles in metabolic switching and adaptation by modifying metabolic enzymes and transcription factors [119,120]. For instance, acetylation of the transcription factor FOXO1 can alter its DNA-binding capacity, transcriptional activity, and subcellular localization. In kidney disease, the acetylation status of FOXO1 affects its ability to regulate genes involved in apoptosis, autophagy, and oxidative stress [121,122,123]. Crystal injury may remodel the acetylation profile of transcription factors like FOXO1 by altering the activity of acetyltransferases or deacetylases, thereby shifting cellular fate decisions [35,124,125]. At the level of epigenetics and signal transduction, the histone methyltransferase SMYD2 is upregulated in stone-bearing kidney tissue. It methylates the PTEN protein, relieving its inhibition on the PI3K/AKT/mTOR pathway. This activates the pathway, driving metabolic reprogramming of renal tubular cells toward glycolysis, which promotes apoptosis, inflammation, epithelial–mesenchymal transition, and consequently, stone formation [46]. Similarly, Protein Arginine Methyltransferase 1 (PRMT1) can methylate the ubiquitin-conjugating enzyme UBE2m, enhancing its function. This leads to the ubiquitination and degradation of the transcription factor PPARγ, causing renal lipid accumulation and energy metabolism disorder [42]. Metabolites can also serve as substrates for PTMs to influence gene expression. For example, Lgals3 expression is elevated in the stone environment. It promotes glycolysis and lactate production by stabilizing the glycolytic key enzyme PKM2 (inhibiting its ubiquitination degradation). Lactate, in turn, induces lactylation modification at the histone H3K18 site, activating the transcription of pro-lithogenic and injury-related genes such as FGFR4 [37].

It is noteworthy that our preliminary research found that the protective deacetylase Sirt6 undergoes ubiquitination and is degraded via the autophagy-lysosome pathway under stone-forming conditions. Its decreased level impairs DNA damage repair capacity, exacerbating cell injury. Stabilizing or activating Sirt6 promotes DNA repair, restoring the balance between damage and repair [39]. These findings reveal how PTMs regulate cellular metabolic states through multi-layered networks, enabling adaptation to or worsening of the stone microenvironment, ultimately influencing disease outcome.

Importantly, the spectrum of PTMs implicated in nephrolithiasis may extend well beyond those discussed above. In other kidney diseases, SUMOylation and NEDDylation have been confirmed to regulate inflammatory responses and protein stability—key factors in crystal-induced cellular injury. However, the roles of these modification types in the context of nephrolithiasis remain unknown, and future research should investigate them thoroughly within the framework of stone pathogenesis.

## 4. Interplay and Regulatory Networks of Protein Post-Translational Modifications

**Figure 2:**
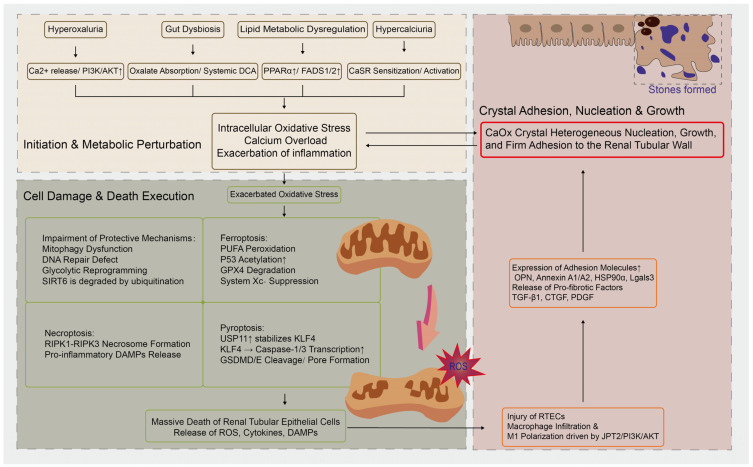
The Role of Post-translational Modifications in the Pathogenesis of Nephrolithiasis-Associated Kidney Injury. Black arrows (→) indicate direct regulation or progression; upward arrows (↑) denote increased expression/activity; different shapes (rectangles, rounded rectangles) are used to distinguish between pathophysiological conditions, cellular processes, and molecular events.

PTMs constitute a multidimensional and dynamic regulatory network throughout the process of kidney stone formation. Their action begins with the initiation of crystal–cell adhesion, where diverse modifications alter cell surface properties and signal transduction, thereby creating the initial conditions for crystal retention. Subsequently, PTMs function as pivotal switches, precisely regulating the amplification of inflammatory and oxidative stress cascades, as well as determining cell fate decisions. Ultimately, through extensive modifications of metabolic enzymes and transcription factors, PTMs drive cellular metabolic reprogramming, promoting either adaptation to or exacerbation of the pathological microenvironment. These mechanisms do not operate in isolation; instead, they are tightly interwoven through “cross-talk,” integrating external crystal-derived signals into a core cellular response network that dictates the balance between injury and repair. This integrated framework provides a critical molecular basis for a deeper understanding of stone pathogenesis and for the development of targeted intervention strategies (Figure 2).

The realization of this multidimensional regulatory function fundamentally depends on how protein functions are executed. Protein function is not carried out in isolation but is highly dependent on the complex networks of protein–protein interactions (PPI) formed within cells [126,127]. PTMs serve as one of the key mechanisms regulating PPI, either by directly creating or blocking binding sites or by indirectly influencing interactions through alterations in protein conformation [128].

Research in the field of nephrolithiasis reveals that ER stress and oxidative stress, triggered by CaOx crystal stimulation, act as central hubs initiating the dynamic interactive network of PTMs. This network precisely regulates cellular injury and repair processes through the synergy and antagonism of various modifications, including ubiquitination, phosphorylation, acetylation, and glycosylation [129]. Crystal injury can induce the phosphorylation of receptor tyrosine kinases, such as the epidermal growth factor receptor, to activate cell survival signals [32], while simultaneously potentially affecting the stability of nuclear proteins through modifications like SUMOylation [130]. For example, Lgals3 promotes H3K18 lactylation while inhibiting the ubiquitination of PKM2, thereby contributing to stone formation. At the level of inflammation and fibrosis, cytokines activate the OSMRβ/STAT3 phosphorylation signaling axis, upregulating the expression of crystal-binding molecules (e.g., osteopontin, annexins) and pro-fibrotic factors [32]. OPN, as a key regulatory protein, has its own function intricately modulated by complex PTMs such as phosphorylation, sulfation, and O-linked glycosylation [131]. ER stress not only directly induces the unfolded protein response and apoptosis but can also indirectly interfere with the proper folding and modification of crucial glycoproteins like OPN and THP by affecting chaperone function and calcium homeostasis, thereby altering their inhibitory or promotive effects on crystal formation [70]. Furthermore, findings from other renal fibrosis models indicate that lipid modifications, such as palmitoylation, can regulate the stability and localization of key signaling proteins like β-catenin, suggesting that similar cross-talk among modifications may exist in the context of stone disease [132]. These modification events do not operate in a linear fashion but constitute a complex “cross-talk” network. For instance, phosphorylation can provide recognition sites for ubiquitination; acetylation and methylation collectively determine chromatin states; and different modifications form cascades by sharing target proteins or competing with each other. Ultimately, this integrated network translates an external signal of crystal injury into a decision-making network determining cellular fate [133].

## 5. Clinical Translation Potential and Future Perspectives

In recent years, rapid advancements in proteomic technologies, such as mass spectrometry, have empowered researchers to systematically identify and quantitatively analyze protein PTMs, providing a powerful tool for discovering novel disease biomarkers and drug targets [134,135].

Nephrolithiasis is a disease characterized by high incidence and recurrence rates, with a complex pathogenesis involving the dynamic equilibrium of multiple promotive and inhibitory factors. Traditional diagnosis and risk assessment primarily rely on urine chemistry analysis, but its predictive capacity is limited [136,137]. Therefore, identifying more precise biomarkers for predicting stone risk, monitoring recurrence, and developing novel therapeutic strategies is crucial. Research indicates that urinary proteins play a dual role in nephrolithiasis, capable of either inhibiting or promoting crystal nucleation, growth, and aggregation [131]. The function of these proteins is largely regulated by their PTMs [60]. Consequently, in-depth investigation into the PTM patterns of proteins associated with kidney stones not only aids in elucidating the disease mechanisms but also opens new avenues for developing innovative biomarkers and therapeutic targets.

### 5.1. PTMs as Biomarkers: Challenges and Opportunities

Translating the detection of PTMs into clinically applicable biomarkers presents several challenges: (1) The dynamic nature of PTMs: Modification levels can fluctuate rapidly with physiological states, making it difficult for a single measurement to reflect the true situation; (2) Low abundance: Modified proteins often constitute only a small fraction of the total protein pool, necessitating high detection sensitivity; (3) Site specificity: Modifications at different sites on the same protein can have opposing functions, requiring precise quantification. Potential strategies to address these challenges include developing highly sensitive and specific targeted mass spectrometry methods, establishing standardized sample processing protocols, integrating machine learning algorithms to identify stable PTM signature profiles, and conducting longitudinal cohort studies to validate the predictive value of PTM biomarkers.

### 5.2. Therapeutic Prospects of Targeting PTM Enzymes

Although no direct PTM-targeted drug studies for nephrolithiasis currently exist, research in other disease areas provides a theoretical foundation for this strategy. Several reviews highlight that small-molecule inhibitors or activators targeting PTM-regulating enzymes—such as kinases, methyltransferases, and deacetylases—have achieved significant success in oncology, metabolic diseases, and other fields [138]. For instance, in CKD research, PTMs like phosphorylation and ubiquitination within the TGF-β/Smad signaling pathway are recognized as key regulatory nodes. Targeting these PTMs holds promise for developing innovative anti-fibrotic therapies [139].

In the field of nephrolithiasis, the most promising targets include the following: (1) Sirt1 activators: Sirt1 inhibits oxidative stress, inflammation, and ferroptosis by deacetylating substrates such as p53 and FOXO1, with protective effects confirmed in animal studies [35]; (2) HDAC2 inhibitors: Although there are currently no reports on the use of HDAC2 inhibitors specifically for stone treatment, multiple studies suggest that HDAC inhibitors can reduce renal tubular epithelial cell apoptosis and fibrosis—processes that are highly consistent with the pathogenesis and progression of nephrolithiasis [117,118]; (3) AMPK activators: These agents exert protective effects by promoting autophagy and suppressing inflammation [27]; (4) Specific kinase inhibitors: For example, inhibitors of the STAT3 pathway can reduce the expression of crystal adhesion molecules and pro-inflammatory factors [26]. However, the off-target effects of these intervention strategies cannot be overlooked. Broad-spectrum HDAC inhibitors, for instance, may affect the cell cycle and gene expression, leading to adverse reactions [140]. Therefore, the development of highly selective small-molecule compounds, or the use of nano-delivery systems to achieve renal targeting, may represent important future research directions.

### 5.3. Limitations and Future Perspectives

This review systematically summarizes the role of PTMs in the pathogenesis of nephrolithiasis; however, several limitations should be acknowledged. First, the current evidence is derived primarily from in vitro studies and animal models, with a relative lack of human validation data. Second, some mechanistic studies have only observed correlations and lack causal evidence. Third, PTM detection techniques have not yet been standardized, which limits the comparability of results across different studies. Fourth, discussions regarding the interplay between different PTMs remain largely speculative and require further direct experimental validation.

To advance the field, we propose the following research priorities: (1) Conduct cohort studies to collect urine samples from stone patients at different stages for PTMs analysis, identifying PTMs signatures associated with stone formation and recurrence. (2) Utilize CRISPR-Cas9 technology to establish animal models with PTMs enzyme gene knockouts/knock-ins to clarify the causal roles of specific PTMs in stone pathogenesis. (3) Develop highly sensitive and high-throughput PTMs detection methods to achieve precise quantification at single-site resolution. (4) Initiate clinical trials to evaluate the safety and efficacy of drugs targeting PTMs enzymes in the prevention and treatment of nephrolithiasis.

## 6. Conclusions

In summary, PTMs may serve as a critical hub in the pathogenesis of nephrolithiasis, dynamically linking crystal-induced physicochemical stress to complex biological responses. PTMs actively regulate a series of key events, from initial crystal–cell adhesion to oxidative stress, inflammatory responses, and the determination of renal tubular cell fate through apoptosis, pyroptosis, and ferroptosis. These modifications form an intricate network—a “PTMs code”—that integrates microenvironmental signals to modulate the balance between renal injury and repair.

This evolving theoretical framework holds significant potential for clinical translation. Targeting specific PTM-regulating enzymes represents a promising new therapeutic strategy, while distinct PTM signatures on urinary proteins may serve as biomarkers for risk stratification. Future research integrating multi-omics approaches with functional validation will be essential to fully leverage these insights for advancing precision medicine in nephrolithiasis.

## Figures and Tables

**Table 1:** Targets for post-translational modification related to kidney stones.

Modification Type	Enzyme	Substrate Protein & Change	Biological Function	Evidence ^1^	Reference
Phosphorylation	PKC ζ	Phosphatidylethanolamine-binding protein 1 is phosphorylated	Exacerbates lipid peroxidation and promotes ferroptosis.	V	[24]
	Akt	Not identified	Promoting crystal–cell adhesion, and modulating macrophage metabolism and inflammatory polarization.	V + I	[25]
	PKA	STAT3 is phosphorylated	Upregulating the expression of claudin-14, thereby promoting the formation of calcium salt kidney stones.	V + I	[26]
	AMPK	Increased phosphorylation level of Nrf2	Inhibiting oxidative stress and inflammatory responses.	V + I	[27]
	AMPK	Increased LC3B-II and accumulated p62	Inhibit the formation of calcium oxalate kidney stones.	I	[28]
	AMPK	Enhance the PINK1-Parkin pathway	Restore impaired mitophagy and inhibit oxidative stress, inflammation, and ferroptosis.	V + I	[29]
	RIPK3	Block the formation of the RIPK1-RIPK3 necrosome complex	Inhibit cellular injury and inflammatory response and reduce intrarenal crystal deposition.	V + I	[30]
	AKT/mTOR	Decreased autophosphorylation levels of PI3K, AKT, and mTOR	Inhibit oxidative stress and calcium ion deposition and increase cell viability.	V + I	[31]
	Not identified	STAT3 is phosphorylated and activated	Inhibit the expression of crystal-binding molecules and inflammation- and fibrosis-associated molecules.	V + I	[32]
Acetylation	Histone acetyltransferase	Histone H3 hyperacetylation	Upregulation of renal CaSR and intestinal calcium transporters promotes hypercalciuria.	I	[33]
Deacetylation	Sirtuin 1	The acetylation of p53 is enhanced.	Inhibits ferroptosis and alleviates calcium oxalate crystal-induced renal fibrosis.	V + I	[34]
	Sirtuin 1	Not identified	Increases itaconate levels and decreases succinate oxidation levels.	V + I	[35]
	Histone Deacetylase 3	Not identified	Renal interstitial fibrosis and aggravated pathological damage.	I	[36]
Lactylation	Not identified	Histone H3 lysine 18 undergoes lactylation	Promotes FGFR4 transcription and expression, which in turn mediates increased deposition of calcium oxalate crystals.	V + I	[37]
	Not identified	Lactylation of lysine 52 on aldehyde dehydrogenase 2	Worsens mitochondrial dysfunction and intensifies renal tubular damage.	V + I	[38]
Ubiquitination	Not identified	The SIRT6 protein undergoes ubiquitination modification	DNA damage repair capacity is weakened, and crystal deposition is increased.	V + I	[39]
	Not identified	GPX4 is ubiquitinated and degraded	Induces cellular ferroptosis and exacerbates calcium oxalate crystal deposition.	V + I	[40]
	Trim21	PKM2 is ubiquitinated and degraded	Promotes glycolysis inhibition and reduces lactate production, alleviating CaOx crystal formation and renal fibrosis.	V + I	[37]
	VHL protein complex	Cargo adaptor BICD2 undergoes K48-linked polyubiquitination	Inhibits STAT1 nuclear translocation, attenuates IFNγ signaling, and limits oxalate-induced lipid peroxidation and ferroptosis.	V + I	[41]
	NEDD4	PPARγ undergoes ubiquitination modification	Renal lipid accumulation, disruption of energy metabolism, and impairment of renal function.	V + I	[42]
Deubiquitination	USP11	The transcription factor KLF4 undergoes deubiquitination	Enhances renal tubular epithelial cell pyroptosis and promotes inflammation and renal fibrosis.	V + I	[43]
	USP14	Transcription factor AP-2 alpha undergoes deubiquitination modification	Inhibits mitophagy and exacerbates renal injury, oxidative stress, and inflammation.	V + I	[44]
Glycosylation	Not identified	The glycosylation status of plasma-derived prothrombin fragment 1 was altered	Promotes crystal nucleation and inhibits crystal aggregation.	V	[45]
Methylation	PRMT1	Ubiquitin-conjugating enzyme E2 M undergoes arginine methylation at residue R169	Inhibits downstream fatty acid metabolism, leading to renal lipid accumulation.	V + I	[42]
	Methyltransferase SMYD2	Phosphatase and tensin homolog undergo methylation	Induces cell apoptosis, inflammation, and epithelial–mesenchymal transition.	V + I	[46]

Evidence ^1^: V, in vitro (cell experiments); I, in vivo (animal experiments).

## Data Availability

No new data were created or analyzed in this study.

## Abbreviations

The following abbreviations are used in this manuscript:

PTMs	Post-translational modifications
CKD	Chronic kidney disease
CaOx	Calcium Oxalate
ER	Endoplasmic Reticulum
ROS	Reactive Oxygen Species
OPN	Osteopontin
OSM	Oncostatin M
IGF1R	Insulin-like Growth Factor 1 Receptor
HSP90	Heat Shock Protein 90
THP	Tamm-Horsfall Protein
PTF1	Prothrombin fragment 1
Sirt1	Sirtuin 1
Sirt6	Sirtuin 6
PRMT1	Protein Arginine Methyltransferase 1
HDAC3	Histone Deacetylase 3
DCA	Deoxycholic Acid
VDR	Vitamin D Receptor
VHL	Von Hippel-Lindau
PPI	Protein–protein interaction

## References

[1] Bargagli M., Scoglio M., Howles S.A., Fuster D.G. (2025). Kidney stone disease: Risk factors, pathophysiology and management. Nat. Rev. Nephrol..

[2] Zhong Y., Zeng Q., Yi J., Long T., Peng J., Zhong L. (2024). Research trends and frontiers on risk factors of urinary stones: A bibliometric analysis from 2010 to 2023. Ren. Fail..

[3] Siener R. (2021). Nutrition and Kidney Stone Disease. Nutrients.

[4] Li S., Huang X., Liu J., Yue S., Hou X., Hu L., Wu J. (2022). Trends in the Incidence and DALYs of Urolithiasis from 1990 to 2019: Results from the Global Burden of Disease Study 2019. Front. Public Health.

[5] Wang Z., Zhang Y., Zhang J., Deng Q., Liang H. (2021). Recent advances on the mechanisms of kidney stone formation (Review). Int. J. Mol. Med..

[6] Dror I., Merlin C., Shilo Y., Berkowitz B. (2025). Linking basic principles of solution chemistry to kidney stone formation timelines. Sci. Rep..

[7] Eren E., Karabulut Y.Y., Eren M., Kadir S. (2023). Mineralogy, geochemistry, and micromorphology of human kidney stones (urolithiasis) from Mersin, the southern Turkey. Environ. Geochem. Health.

[8] Ferraro P.M., Taylor E.N., Curhan G.C. (2024). 24-Hour Urinary Chemistries and Kidney Stone Risk. Am. J. Kidney Dis..

[9] Awuah Boadi E., Shin S., Gombedza F., Bandyopadhyay B.C. (2021). Differential biomolecular recognition by synthetic vs. biologically-derived components in the stone-forming process using 3D microfluidics. J. Mater. Chem. B.

[10] Dong C., Zhou J., Su X., He Z., Song Q., Song C., Ke H., Wang C., Liao W., Yang S. (2024). Understanding formation processes of calcareous nephrolithiasis in renal interstitium and tubule lumen. J. Cell. Mol. Med..

[11] Tamborino F., Cicchetti R., Mascitti M., Litterio G., Orsini A., Ferretti S., Basconi M., De Palma A., Ferro M., Marchioni M. (2024). Pathophysiology and Main Molecular Mechanisms of Urinary Stone Formation and Recurrence. Int. J. Mol. Sci..

[12] Dutta H., Jain N. (2023). Post-translational modifications and their implications in cancer. Front. Oncol..

[13] Zhong Q., Xiao X., Qiu Y., Xu Z., Chen C., Chong B., Zhao X., Hai S., Li S., An Z. (2023). Protein posttranslational modifications in health and diseases: Functions, regulatory mechanisms, and therapeutic implications. MedComm.

[14] Wu X., Xu M., Geng M., Chen S., Little P.J., Xu S., Weng J. (2023). Targeting protein modifications in metabolic diseases: Molecular mechanisms and targeted therapies. Signal Transduct. Target. Ther..

[15] Bashyal A., Brodbelt J.S. (2024). Uncommon posttranslational modifications in proteomics: ADP-ribosylation, tyrosine nitration, and tyrosine sulfation. Mass Spectrom. Rev..

[16] Zhang H., Yan Q., Jiang S., Hu D., Lu P., Li S., Sandai D., Zhang H., Zhang W., Zhu C. (2025). Protein post-translational modifications and tumor immunity: A pan-cancer perspective. Phys. Life Rev..

[17] Hassanzadeh K., Liu J., Maddila S., Mouradian M.M. (2024). Posttranslational Modifications of α-Synuclein, Their Therapeutic Potential, and Crosstalk in Health and Neurodegenerative Diseases. Pharmacol. Rev..

[18] Li Z., Zhu T., Wu Y., Yu Y., Zang Y., Yu L., Zhang Z. (2025). Functions and mechanisms of non-histone post-translational modifications in cancer progression. Cell Death Discov..

[19] Bi B., Qiu M., Liu P., Wang Q., Wen Y., Li Y., Li B., Li Y., He Y., Zhao J. (2023). Protein post-translational modifications: A key factor in colorectal cancer resistance mechanisms. Biochim. Biophys. Acta Gene Regul. Mech..

[20] Liu Z., Yang J., Du M., Xin W. (2023). Functioning and mechanisms of PTMs in renal diseases. Front. Pharmacol..

[21] Wei M., Lin J., Zeng Y., Wang X., Wen J., Wang J., Zou W., Tu K., Liu M., Li J. (2025). Enzymatic post-translational modifications of proteins in chronic kidney disease: Mechanisms, regulation, and clinical significance. Front. Pharmacol..

[22] Laget J., Duranton F., Argilés À., Gayrard N. (2022). Renal insufficiency and chronic kidney disease—Promotor or consequence of pathological post-translational modifications. Mol. Asp. Med..

[23] Chen T., Qian B., Zou J., Luo P., Zou J., Li W., Chen Q., Zheng L. (2023). Oxalate as a potent promoter of kidney stone formation. Front. Med..

[24] Wang R., Zhang J., Ren H., Qi S., Xie L., Xie H., Shang Z., Liu C. (2024). Dysregulated palmitic acid metabolism promotes the formation of renal calcium-oxalate stones through ferroptosis induced by polyunsaturated fatty acids/phosphatidic acid. Cell. Mol. Life Sci..

[25] Song Q., Song C., Chen X., Xiong Y., He Z., Su X., Zhou J., Ke H., Dong C., Liao W. (2024). Oxalate regulates crystal-cell adhesion and macrophage metabolism via JPT2/PI3K/AKT signaling to promote the progression of kidney stones. J. Pharm. Anal..

[26] Luo P., Chen T., Zheng L., Zou J., Zou J., Li W., Chen Q., Cheng L., Qian B. (2024). Calcium sensing receptor regulate claudin-14 via PKA-STAT3 pathway in rat model of nephrolithiasis. Front. Pharmacol..

[27] Tian H., Liang Q., Shi Z., Zhao H. (2023). Hyperoside Ameliorates Renal Tubular Oxidative Damage and Calcium Oxalate Deposition in Rats through AMPK/Nrf2 Signaling Axis. J. Renin-Angiotensin-Aldosterone Syst..

[28] Liu C.J., Ho K.T., Huang H.S., Lu Z.H., Hsieh M.H., Chang Y.S., Wang W.H., Lai E.C., Tsai Y.S. (2025). Sodium glucose co-transporter 2 inhibitor prevents nephrolithiasis in non-diabetes by restoring impaired autophagic flux. eBioMedicine.

[29] Zhou J., Meng L., He Z., Song Q., Liu J., Su X., Wang C., Ke H., Dong C., Liao W. (2023). Melatonin exerts a protective effect in ameliorating nephrolithiasis via targeting AMPK/PINK1-Parkin mediated mitophagy and inhibiting ferroptosis in vivo and in vitro. Int. Immunopharmacol..

[30] Hou B., Liu M., Chen Y., Ni W., Suo X., Xu Y., He Q., Meng X., Hao Z. (2022). Cpd-42 protects against calcium oxalate nephrocalcinosis-induced renal injury and inflammation by targeting RIPK3-mediated necroptosis. Front. Pharmacol..

[31] Zheng X., Lv S., Wang W., Zhu L., Lin L. (2026). Lysimachia christinae Hance aqueous extract ameliorates renal injury in kidney stone rats and calcium oxalate crystal-induced oxidative stress in HK-2 cells via inhibiting the PI3K/Akt/mTOR pathway. Histol. Histopathol..

[32] Deguchi R., Komori T., Yamashita S., Hisaoka T., Kajimoto M., Kohjimoto Y., Hara I., Morikawa Y. (2024). Suppression of renal crystal formation, inflammation, and fibrosis by blocking oncostatin M receptor β signaling. Sci. Rep..

[33] Guo S., Chia W., Wang H., Bushinsky D.A., Zhong B., Favus M.J. (2022). Vitamin D receptor (VDR) contributes to the development of hypercalciuria by sensitizing VDR target genes to vitamin D in a genetic hypercalciuric stone-forming (GHS) rat model. Genes Dis..

[34] Ye Z., Xia Y., Li L., Li B., Chen L., Yu W., Ruan Y., Rao T., Zhou X., Cheng F. (2023). p53 deacetylation alleviates calcium oxalate deposition-induced renal fibrosis by inhibiting ferroptosis. Biomed. Pharmacother..

[35] Yao X., Liu H., Duan C., Zhang Y., Wu X., Li B., Li S., Gong Y., Liu T., Wang X. (2025). Sirtuin1 mitigation of calcium oxalate nephropathy via enhancing itaconate abundance through reduction of histone trimethylation. Clin. Transl. Med..

[36] Hu L., Yang K., Mai X., Wei J., Ma C. (2022). Depleted HDAC3 attenuates hyperuricemia-induced renal interstitial fibrosis via miR-19b-3p/SF3B3 axis. Cell Cycle.

[37] Ye Z., Sun Y., Yang S., Li L., Li B., Xia Y., Yuan T., Yu W., Chen L., Zhou X. (2025). Lgals3 Promotes Calcium Oxalate Crystal Formation and Kidney Injury Through Histone Lactylation-Mediated FGFR4 Activation. Adv. Sci..

[38] Li J., Shi X., Xu J., Wang K., Hou F., Luan X., Chen L. (2025). Aldehyde Dehydrogenase 2 Lactylation Aggravates Mitochondrial Dysfunction by Disrupting PHB2 Mediated Mitophagy in Acute Kidney Injury. Adv. Sci..

[39] Wu W., Li X., Amier Y., Wan W., Yang J., Huang Y., Li J., Yuan D., Zhang J., Zhang X. (2026). SIRT6 Protects renal tubular epithelial cells from hyperoxaluria. Biochem. Pharmacol..

[40] Liu L., Ma Y., Jian Z., Liao B., Li Y., Lin L., Wang M., Chen J., Wei J., Yang M. (2025). Gut microbiota-bile acid crosstalk contributes to calcium oxalate nephropathy through Hsp90α-mediated ferroptosis. Cell Rep..

[41] Hao W., Zhang H., Hong P., Zhang X., Zhao X., Ma L., Qiu X., Ping H., Lu D., Yin Y. (2023). Critical role of VHL/BICD2/STAT1 axis in crystal-associated kidney disease. Cell Death Dis..

[42] Yuan T., Ye Z., Mei S., Zhang M., Wu M., Lin F., Yu W., Li W., Zhou X., Cheng F. (2025). PRMT1-mediated methylation of UBE2m promoting calcium oxalate crystal-induced kidney injury by inhibiting fatty acid metabolism. Cell Death Dis..

[43] Wang X., Xie X., Ni J.Y., Li J.Y., Sun X.A., Xie H.Y., Yang N.H., Guo H.J., Lu L., Ning M. (2025). USP11 promotes renal tubular cell pyroptosis and fibrosis in UUO mice via inhibiting KLF4 ubiquitin degradation. Acta Pharmacol. Sin..

[44] Li Y., Dong B., Wang Y., Bi H., Zhang J., Ding C., Wang C., Ding X., Xue W. (2024). Inhibition of Usp14 ameliorates renal ischemia-reperfusion injury by reducing Tfap2a stabilization and facilitating mitophagy. Transl. Res..

[45] Webber D., Rodgers A.L., Sturrock E.D. (2007). Glycosylation of prothrombin fragment 1 governs calcium oxalate crystal nucleation and aggregation, but not crystal growth. Urol. Res..

[46] Pan S., Yuan T., Xia Y., Yu W., Li H., Rao T., Ye Z., Li L., Zhou X., Cheng F. (2024). SMYD2 Promotes Calcium Oxalate-Induced Glycolysis in Renal Tubular Epithelial Cells via PTEN Methylation. Biomedicines.

[47] Lai Y., Zheng H., Sun X., Lin J., Li Q., Huang H., Hou Y., Zhong H., Zhang D., Fucai T. (2022). The advances of calcium oxalate calculi associated drugs and targets. Eur. J. Pharmacol..

[48] Yoodee S., Peerapen P., Thongboonkerd V. (2024). Defining physicochemical properties of urinary proteins that determine their inhibitory activities against calcium oxalate kidney stone formation. Int. J. Biol. Macromol..

[49] Ying X., Chen Y., Hao Z., Liu H. (2025). The significance of reactive oxygen species in the formation of calcium oxalate stones and the protective effects of antioxidants on the kidneys. Front. Immunol..

[50] Khan S.R., Canales B.K., Dominguez-Gutierrez P.R. (2021). Randall’s plaque and calcium oxalate stone formation: Role for immunity and inflammation. Nat. Rev. Nephrol..

[51] Dong C., He Z., Liao W., Jiang Q., Song C., Song Q., Su X., Xiong Y., Wang Y., Meng L. (2025). CHAC1 Mediates Endoplasmic Reticulum Stress-Dependent Ferroptosis in Calcium Oxalate Kidney Stone Formation. Adv. Sci..

[52] Li Y., Yang B., Wang H., Hu W., Liu T., Lu X., Gao B. (2025). CAV1 unveils a novel therapeutic target for nephrolithiasis by modulating CaSR and ER stress. Biochim. Biophys. Acta Mol. Basis Dis..

[53] Hong S.Y., Miao L.T., Qin B.L. (2024). The Involvement of Endoplasmic Reticulum Stress during the Interaction between Calcium Oxalate Crystals and Renal Tubular Epithelial Cells. Biology.

[54] Yang B., Lu X., Li Y., Li Y., Yu D., Zhang W., Duan C., Taguchi K., Yasui T., Kohri K. (2019). A Proteomic Network Approach across the Kidney Stone Disease Reveals Endoplasmic Reticulum Stress and Crystal-Cell Interaction in the Kidney. Oxidative Med. Cell. Longev..

[55] Hong S.Y., Qin B.L. (2024). The Altered Proteomic Landscape in Renal Tubular Epithelial Cells under High Oxalate Stimulation. Biology.

[56] Lu C.L., Tseng Y.S., Wu W.B., Liao C.H., Ma M.C. (2025). Hydrogen Sulfide Deficiency Contributes to Tubular Damage and Calcium Oxalate Crystal Formation in Hyperoxaluria Nephropathy: Role of Osteopontin and Tamm-Horsfall Protein. Antioxidants.

[57] He Z., Dong C., Song T., Zhou J., Xu T., He R., Li S. (2024). FTH1 overexpression using a dCasRx translation enhancement system protects the kidney from calcium oxalate crystal-induced injury. Cell. Mol. Biol. Lett..

[58] Bai J., Lu T., Zhao Z., Wei F., He K., He X., Jing Y., Wang F., Qin W., Xu Z. (2025). The role of inflammation and ROS in CaOx kidney stones. Eur. J. Med. Res..

[59] Xiang H., Yang Y., Chen L., He Z., Zhou Q., Dong C., Jiang Q., Chen Q., Su X., Yang S. (2025). Cadmium exposure upregulates TXNIP and aggravates calcium oxalate kidney stone formation by promoting cell-crystal adhesion, apoptosis and macrophage M1 polarization. Ecotoxicol. Environ. Saf..

[60] Hadpech S., Peerapen P., Chaiyarit S., Sritippayawan S., Thongboonkerd V. (2026). Urinary proteins from stone formers promote calcium oxalate crystallization, growth and aggregation via oxidative modifications. J. Adv. Res..

[61] Averill-Bates D. (2024). Reactive oxygen species and cell signaling. Review. Biochim. Biophys. Acta Mol. Cell Res..

[62] Niekerk L.A., Gokul A., Basson G., Badiwe M., Nkomo M., Klein A., Keyster M. (2024). Heavy metal stress and mitogen activated kinase transcription factors in plants: Exploring heavy metal-ROS influences on plant signalling pathways. Plant Cell Environ..

[63] Bu S., Zhang T., Xu H., Huang X., He P., Gao J., Liu R. (2025). Associations of metabolic syndrome and its components with complex renal calculi and stone composition: A cross-sectional study. Eur. J. Med. Res..

[64] Kong H., Han J., Guo L., Zhang X.A. (2026). Targeting post-translational modifications: Novel insights into bone metabolic diseases. J. Adv. Res..

[65] Yang W., Wu J., Cui G. (2026). Metabolites beyond metabolism: Exploring their atypical roles in protein modification and signaling transduction. Chin. Med. J..

[66] Wang J., Zhao Q., Zhang S., Liu J., Fan X., Han B., Hou Y., Ai X. (2026). Microbial short chain fatty acids: Effective histone deacetylase inhibitors in immune regulation (Review). Int. J. Mol. Med..

[67] Yu Y., Moretti I.F., Grzeschik N.A., Sibon O.C.M., Schepers H. (2021). Coenzyme A levels influence protein acetylation, CoAlation and 4’-phosphopantetheinylation: Expanding the impact of a metabolic nexus molecule. Biochim. Biophys. Acta Mol. Cell Res..

[68] Jia Q., Huang Z., Wang G., Sun X., Wu Y., Yang B., Yang T., Liu J., Li P., Li J. (2022). Osteopontin: An important protein in the formation of kidney stones. Front. Pharmacol..

[69] Lang F., Li Y., Yao R., Jiang M. (2025). Osteopontin in Chronic Inflammatory Diseases: Mechanisms, Biomarker Potential, and Therapeutic Strategies. Biology.

[70] Bhardwaj R., Bhardwaj A., Dhawan D.K., Tandon C., Kaur T. (2022). 4-PBA rescues hyperoxaluria induced nephrolithiasis by modulating urinary glycoproteins: Cross talk between endoplasmic reticulum, calcium homeostasis and mitochondria. Life Sci..

[71] Yamashita S., Komori T., Kohjimoto Y., Miyajima A., Hara I., Morikawa Y. (2020). Essential roles of oncostatin M receptor β signaling in renal crystal formation in mice. Sci. Rep..

[72] Pan J., Zhang Y., Yao R., Yang M., Mao X., Song Z., Xu Y., Chen Y., Hou B., Liu X. (2025). IGF1R Enhances Calcium Oxalate Monohydrate-Induced Epithelial-Mesenchymal Transition by Reprogramming Metabolism via the JAK2/STAT3 Signaling. Int. J. Biol. Sci..

[73] Yoodee S., Peerapen P., Plumworasawat S., Thongboonkerd V. (2022). Roles of heat-shock protein 90 and its four domains (N, LR, M and C) in calcium oxalate stone-forming processes. Cell. Mol. Life Sci..

[74] Hadpech S., Peerapen P., Rattananinsruang P., Detsangiamsak S., Phuangkham S., Chotikawanich E., Sritippayawan S., Thongboonkerd V. (2025). Comprehensive identification of stone-promoting proteins in the urine of kidney stone formers. Int. J. Biol. Macromol..

[75] Koeipudsa N., Sassanarakkit S., Peerapen P., Thongboonkerd V. (2025). Characteristics of kidney stone-modulatory proteins decoded from proteins identified in stone matrix and urine of stone formers and non-stone subjects. Comput. Struct. Biotechnol. J..

[76] Noonin C., Peerapen P., Yoodee S., Kapincharanon C., Kanlaya R., Thongboonkerd V. (2022). Systematic analysis of modulating activities of native human urinary Tamm-Horsfall protein on calcium oxalate crystallization, growth, aggregation, crystal-cell adhesion and invasion through extracellular matrix. Chem.-Biol. Interact..

[77] Nishio S., Hatanaka M., Takeda H., Aoki K., Iseda T., Iwata H., Yokoyama M. (2001). Calcium phosphate crystal-associated proteins: Alpha-2-HS-glycoprotein, prothrombin fragment 1 and osteopontin. Int. J. Urol..

[78] Song B.F., Li B.J., Ning J.Z., Xia Y.Q., Ye Z.H., Yuan T.H., Yan X.Z., Li L., Zhou X.J., Rao T. (2023). Overexpression of sirtuin 1 attenuates calcium oxalate-induced kidney injury by promoting macrophage polarization. Int. Immunopharmacol..

[79] Yang X., Feng J., Liang W., Zhu Z., Chen Z., Hu J., Yang D., Ding G. (2021). Roles of SIRT6 in kidney disease: A novel therapeutic target. Cell. Mol. Life Sci..

[80] Hadpech S., Chaiyarit S., Thongboonkerd V. (2023). Calcineurin B inhibits calcium oxalate crystallization, growth and aggregation via its high calcium-affinity property. Comput. Struct. Biotechnol. J..

[81] Bierschenk D., Papac-Milicevic N., Bresch I.P., Kovacic V., Bettoni S., Dziedzic M., Wetsel R.A., Eschenburg S., Binder C.J., Blom A.M. (2023). C4b-binding protein inhibits particulate- and crystalline-induced NLRP3 inflammasome activation. Front. Immunol..

[82] Sassanarakkit S., Peerapen P., Thongboonkerd V. (2020). StoneMod: A database for kidney stone modulatory proteins with experimental evidence. Sci. Rep..

[83] Griesser E., Vemula V., Mónico A., Pérez-Sala D., Fedorova M. (2021). Dynamic posttranslational modifications of cytoskeletal proteins unveil hot spots under nitroxidative stress. Redox Biol..

[84] Li Y., Zhao J., Yin Y., Zhang C., Zhang Z., Zheng Y. (2023). The Role of STAT3 Signaling Pathway Activation in Subconjunctival Scar Formation after Glaucoma Filtration Surgery. Int. J. Mol. Sci..

[85] Yoodee S., Malaitad T., Plumworasawat S., Thongboonkerd V. (2025). E53, E96, D162, E247 and D322 in Ca^2+^-binding domains of annexin A2 are essential for regulating intracellular [Ca^2+^] and crystal adhesion to renal cells via ERK1/2 and JNK signaling pathways. Arch. Biochem. Biophys..

[86] Jiang Q., Su X., Liao W., He Z., Wang Y., Jiang R., Dong C., Yang S. (2025). Exploring susceptibility and therapeutic targets for kidney stones through proteome-wide Mendelian randomization. Hum. Mol. Genet..

[87] Konya E., Amasaki N., Umekawa T., Iguchi M., Kurita T. (2002). Influence of urinary sialic acid on calcium oxalate crystal formation. Urol. Int..

[88] Takata T., Inoue S., Kunii K., Masauji T., Moriya J., Motoo Y., Miyazawa K. (2025). Advanced Glycation End-Product-Modified Heat Shock Protein 90 May Be Associated with Urinary Stones. Diseases.

[89] Xu Z., Yao X., Duan C., Liu H., Xu H. (2023). Metabolic changes in kidney stone disease. Front. Immunol..

[90] Cui Q., Jia K., Li F., Zheng J., Wang F. (2025). Post-translational modifications in heat stress-related diseases. Front. Mol. Biosci..

[91] Zhang X., Fu Z., Wang H., Sheng L. (2024). Metabolic pathways, genomic alterations, and post-translational modifications in pulmonary hypertension and cancer as therapeutic targets and biomarkers. Front. Pharmacol..

[92] Qi Q., Xu Y., Yu J., Huang Q., Chen Y., Hou B., Hao Z. (2025). Lead exposure aggravates calcium oxalate crystal-induced renal injury and inflammation by upregulating IRF7 and promoting M1 macrophage polarization. Ecotoxicol. Environ. Saf..

[93] Duan C., Li B., Liu H., Zhang Y., Yao X., Liu K., Wu X., Mao X., Wu H., Xu Z. (2024). Sirtuin1 Suppresses Calcium Oxalate Nephropathy via Inhibition of Renal Proximal Tubular Cell Ferroptosis Through PGC-1α-mediated Transcriptional Coactivation. Adv. Sci..

[94] Yan Q., Hu Q., Li G., Qi Q., Song Z., Shu J., Liang H., Liu H., Hao Z. (2023). NEAT1 Regulates Calcium Oxalate Crystal-Induced Renal Tubular Oxidative Injury via miR-130/IRF1. Antioxid. Redox Signal..

[95] Law M., Wang P.C., Zhou Z.Y., Wang Y. (2024). From Microcirculation to Aging-Related Diseases: A Focus on Endothelial SIRT1. Pharmaceuticals.

[96] Singh V., Ubaid S. (2020). Role of Silent Information Regulator 1 (SIRT1) in Regulating Oxidative Stress and Inflammation. Inflammation.

[97] Ye Q.L., Wang D.M., Wang X., Zhang Z.Q., Tian Q.X., Feng S.Y., Zhang Z.H., Yu D.X., Ding D.M., Xie D.D. (2021). Sirt1 inhibits kidney stones formation by attenuating calcium oxalate-induced cell injury. Chem.-Biol. Interact..

[98] Duan C., Liu H., Yang X., Liu J., Deng Y., Wang T., Xing J., Hu Z., Xu H. (2023). Sirtuin1 inhibits calcium oxalate crystal-induced kidney injury by regulating TLR4 signaling and macrophage-mediated inflammatory activation. Cell. Signal..

[99] An S., Yao Y., Hu H., Wu J., Li J., Li L., Wu J., Sun M., Deng Z., Zhang Y. (2023). PDHA1 hyperacetylation-mediated lactate overproduction promotes sepsis-induced acute kidney injury via Fis1 lactylation. Cell Death Dis..

[100] Zuo Q., Lin L., Zhang Y., Ommati M.M., Wang H., Zhao J. (2024). The Footprints of Mitochondrial Fission and Apoptosis in Fluoride-Induced Renal Dysfunction. Biol. Trace Elem. Res..

[101] Liu J., Huang J., Gong B., Cheng S., Liu Y., Chen Y., Feng Q., Li J., Qiu M., Yu G. (2023). Polydatin protects against calcium oxalate crystal-induced renal injury through the cytoplasmic/mitochondrial reactive oxygen species-NLRP3 inflammasome pathway. Biomed. Pharmacother..

[102] Sun X., Gao C., Zhang P., Peng Y., Wang M., Liu J., Ma C., Li S., Xia Z. (2025). Urolithin A protects against calcium oxalate-induced crystal formation and kidney injury by regulating PCK1 to restore mitophagy function in kidney stone disease. Biochim. Biophys. Acta Mol. Basis Dis..

[103] Zhang B., Li F., Shi Y., Ji C., Kong Q., Sun K., Sun X. (2024). Single-cell RNA sequencing integrated with bulk RNA sequencing analysis reveals the protective effects of lactate-mediated lactylation of microglia-related proteins on spinal cord injury. CNS Neurosci. Ther..

[104] Jiang J., Wang R., Song P., Peng Q., Jin X., Li B., Ni J., Shen J., Bao J., Wu Z. (2025). Lactate Facilitates Pancreatic Repair Following Acute Pancreatitis by Promoting Reparative Macrophage Polarization. Cell. Mol. Gastroenterol. Hepatol..

[105] Zhao L., Hao Y., Tang S., Han X., Li R., Zhou X. (2023). Energy metabolic reprogramming regulates programmed cell death of renal tubular epithelial cells and might serve as a new therapeutic target for acute kidney injury. Front. Cell Dev. Biol..

[106] Li Z., Liu Z., Luo M., Li X., Chen H., Gong S., Zhang M., Zhang Y., Liu H., Li X. (2022). The pathological role of damaged organelles in renal tubular epithelial cells in the progression of acute kidney injury. Cell Death Discov..

[107] Tang K., Ye T., He Y., Ba X., Xia D., Peng E., Chen Z., Ye Z., Yang X. (2025). Ferroptosis, necroptosis, and pyroptosis in calcium oxalate crystal-induced kidney injury. Biochim. Biophys. Acta Mol. Basis Dis..

[108] Khan S.R., Alli A.A. (2025). Apoptosis, ferroptosis, necrosis, necroptosis and pyroptosis in the formation of calcium oxalate kidney stones. Urolithiasis.

[109] Lv Y., Wu M., Wang Z., Wang J. (2023). Ferroptosis: From regulation of lipid peroxidation to the treatment of diseases. Cell Biol. Toxicol..

[110] Chen H., Lin X., Yi X., Liu X., Yu R., Fan W., Ling Y., Liu Y., Xie W. (2022). SIRT1-mediated p53 deacetylation inhibits ferroptosis and alleviates heat stress-induced lung epithelial cells injury. Int. J. Hyperth..

[111] Huang D., Liang Y.L., Zhang L.L., Zhou B., Tang B. (2025). Sirtuin 1/3 regulates p53 deacetylation to inhibit iron poisoning-induced alveolar epithelial cell death and contributes to Rapamycin-mediated protection against limb ischemia/reperfusion-induced lung injury. Chem.-Biol. Interact..

[112] Yang H., Park D., Ryu J., Park T. (2021). USP11 degrades KLF4 via its deubiquitinase activity in liver diseases. J. Cell. Mol. Med..

[113] Patra S., Mahapatra K.K., Praharaj P.P., Panigrahi D.P., Bhol C.S., Mishra S.R., Behera B.P., Singh A., Jena M., Bhutia S.K. (2021). Intricate role of mitochondrial calcium signalling in mitochondrial quality control for regulation of cancer cell fate. Mitochondrion.

[114] Zhang K., Fang X., Zhang Y., Zhang Y., Chao M. (2024). Transcriptional activation of PINK1 by MyoD1 mediates mitochondrial homeostasis to induce renal calcification in pediatric nephrolithiasis. Cell Death Discov..

[115] Nie J., Zhang Y., Ning L., Yan Z., Duan L., Xi H., Niu Q., Zhang Q. (2022). Phosphorylation of p53 by Cdk5 contributes to benzo[a]pyrene-induced neuronal apoptosis. Environ. Toxicol..

[116] Ma T., Huang C., Meng X., Li X., Zhang Y., Ji S., Li J., Ye M., Liang H. (2016). A potential adjuvant chemotherapeutics, 18β-glycyrrhetinic acid, inhibits renal tubular epithelial cells apoptosis via enhancing BMP-7 epigenetically through targeting HDAC2. Sci. Rep..

[117] Kong L., Wang H., Li C., Cheng H., Cui Y., Liu L., Zhao Y. (2021). Sulforaphane Ameliorates Diabetes-Induced Renal Fibrosis through Epigenetic Up-Regulation of BMP-7. Diabetes Metab. J..

[118] Ye K., Li J., Huo Z., Xu J., Dai Q., Qiao K., Cao Y., Yan L., Liu W., Hu Y. (2025). Down-regulating HDAC2-LTA4H pathway ameliorates renal ischemia-reperfusion injury. Biochim. Biophys. Acta Mol. Basis Dis..

[119] Li Z., Lu S., Li X. (2021). The role of metabolic reprogramming in tubular epithelial cells during the progression of acute kidney injury. Cell. Mol. Life Sci..

[120] Gao H., Lin J., Xiong F., Yu Z., Pan S., Huang Y. (2022). Urinary Microbial and Metabolomic Profiles in Kidney Stone Disease. Front. Cell. Infect. Microbiol..

[121] Ruan Y., Xue Y., Zhang P., Jia J. (2024). Acetylation of FOXO1 is involved in cadmium-induced rat kidney injury via mediating autophagosome-lysosome fusion blockade and autophagy inhibition. Ecotoxicol. Environ. Saf..

[122] Wang Y., He W. (2021). Improving the Dysregulation of FoxO1 Activity Is a Potential Therapy for Alleviating Diabetic Kidney Disease. Front. Pharmacol..

[123] He Y., Yang X., Zhang C., Deng M., Tu B., Liu Q., Cai J., Zhang Y., Su L., Yang Z. (2025). Ablation of macrophage transcriptional factor FoxO1 protects against ischemia-reperfusion injury-induced acute kidney injury. Acta Pharm. Sin. B.

[124] Cheng Y., Xiao Z., Cai W., Zhou T., Yang Z. (2025). Suppression of FOXO1 activity by SIRT1-mediated deacetylation weakening the intratumoral androgen autocrine function in glioblastoma. Cancer Gene Ther..

[125] Yazıcı E., McIntyre J. (2025). The complex network of p300/CBP regulation: Interactions, posttranslational modifications, and therapeutic implications. J. Biol. Chem..

[126] Suskiewicz M.J. (2024). The logic of protein post-translational modifications (PTMs): Chemistry, mechanisms and evolution of protein regulation through covalent attachments. BioEssays.

[127] Ye H., Han Y., Li P., Su Z., Huang Y. (2022). The Role of Post-Translational Modifications on the Structure and Function of Tau Protein. J. Mol. Neurosci..

[128] Wang S., Osgood A.O., Chatterjee A. (2022). Uncovering post-translational modification-associated protein-protein interactions. Curr. Opin. Struct. Biol..

[129] Nong W.J., Tong X.Y., Ouyang J.M. (2024). Comparison of Endoplasmic Reticulum Stress and Pyroptosis Induced by Pathogenic Calcium Oxalate Monohydrate and Physiologic Calcium Oxalate Dihydrate Crystals in HK-2 Cells: Insights into Kidney Stone Formation. Cells.

[130] Sheng Z., Cao X., Deng Y.N., Zhao X., Liang S. (2023). SUMOylation of AnxA6 facilitates EGFR-PKCα complex formation to suppress epithelial cancer growth. Cell Commun. Signal..

[131] Christensen B., Petersen T.E., Sørensen E.S. (2008). Post-translational modification and proteolytic processing of urinary osteopontin. Biochem. J..

[132] Gu M., Jiang H., Tan M., Yu L., Xu N., Li Y., Wu H., Hou Q., Dai C. (2023). Palmitoyltransferase DHHC9 and acyl protein thioesterase APT1 modulate renal fibrosis through regulating β-catenin palmitoylation. Nat. Commun..

[133] Lacoursiere R.E., Hadi D., Shaw G.S. (2022). Acetylation, Phosphorylation, Ubiquitination (Oh My!): Following Post-Translational Modifications on the Ubiquitin Road. Biomolecules.

[134] Zhai L.H., Chen K.F., Hao B.B., Tan M.J. (2022). Proteomic characterization of post-translational modifications in drug discovery. Acta Pharmacol. Sin..

[135] Pascovici D., Wu J.X., McKay M.J., Joseph C., Noor Z., Kamath K., Wu Y., Ranganathan S., Gupta V., Mirzaei M. (2018). Clinically Relevant Post-Translational Modification Analyses-Maturing Workflows and Bioinformatics Tools. Int. J. Mol. Sci..

[136] Bergsland K.J., Kelly J.K., Coe B.J., Coe F.L. (2006). Urine protein markers distinguish stone-forming from non-stone-forming relatives of calcium stone formers. Am. J. Physiol. Ren. Physiol..

[137] Slocum J.L., Heung M., Pennathur S. (2012). Marking renal injury: Can we move beyond serum creatinine?. Transl. Res..

[138] Cao Y., Yu T., Zhu Z., Zhang Y., Sun S., Li N., Gu C., Yang Y. (2025). Exploring the landscape of post-translational modification in drug discovery. Pharmacol. Ther..

[139] Li J., Zou Y., Kantapan J., Su H., Wang L., Dechsupa N. (2024). TGF-β/Smad signaling in chronic kidney disease: Exploring post-translational regulatory perspectives (Review). Mol. Med. Rep..

[140] Shao R., Suzuki T., Suyama M., Tsukada Y. (2024). The impact of selective HDAC inhibitors on the transcriptome of early mouse embryos. BMC Genom..

